# Increasing obesity odds among foreign-born New Yorkers are not explained by eating out, age at arrival, or duration of residence: results from NYC HANES 2004 and 2013/2014

**DOI:** 10.1186/s12889-021-11351-1

**Published:** 2021-07-26

**Authors:** Margrethe F. Horlyck-Romanovsky, Sean J. Haley

**Affiliations:** 1 Department of Health and Nutrition Sciences, Brooklyn College, City University of New York, 2900 Bedford Avenue, Brooklyn, NY USA; 2 Department of Health Policy and Management, CUNY Graduate School of Public Health and Health Policy, 55 West 125th Street, New York, NY USA

**Keywords:** Obesity, Dietary acculturation, Age at arrival, Duration of residence, Eating out

## Abstract

**Background:**

Among the foreign-born in the United States (US) dietary acculturation and eating out may increase obesity risk.

Using the 2004 (*N* = 1952) and 2013/14 (*N* = 1481) New York City (NYC) Health and Nutrition Examination Surveys, we compared for the foreign-born and US-born by survey year: 1) odds of obesity; 2) association between eating out and obesity and 3) effect of age at arrival and duration of residence among the foreign-born. Weighted logistic regression estimated odds of obesity.

**Results:**

Compared to the US-born, the foreign-born had lower odds of obesity in 2004, (aOR = 0.51 (95%CI 0.37–0.70), *P* = <.0001). Odds were no different in 2013/14. In 2013/14 the foreign-born who ate out had lower obesity odds (aOR = 0.49 (95%CI 0.31–0.77), *P* = 0.0022). The foreign-born living in the US≥10 years had greater odds of obesity in 2004 (aOR = 1.73 (95%CI 1.08–2.79), *P* = 0.0233) but not in 2013/14.

**Conclusions:**

Eating out does not explain increasing obesity odds among the foreign-born.

**Supplementary Information:**

The online version contains supplementary material available at 10.1186/s12889-021-11351-1.

## Introduction

Upon arrival to the United States (US), the foreign-born have historically had healthier eating habits, lower BMI and better cardiometabolic health than the US-born [1–4]. For example, according to the National Health Interview Survey (NHIS) 2000, only 8% of the foreign-born had obesity upon arrival, compared to 22% of the US-born population [4]. However, as the foreign-born settle in the US, access to low-cost, nutrient-dense foods that are richer in calories, sugar, fats, animal protein, and sodium increases [5–7]. In turn, many foreign-born experience significant weight gain over time, such that the foreign-born who have lived in the US for 15 years or more have obesity rates that are no different from the US-born [4]. In another study using 2010–2014 NHIS data**,** duration of residence in the US of more than 15 years among the foreign-born was associated with 19% higher odds of overweight/obesity (Adjusted odds ratio [aOR] 1.19; 95%CI, 1.10–1.29), compared to those living in the US for less than 10 years [1]. According to 2013/14 NHANES data, 37.9% of the total US population had obesity [8].

Changes in food consumption patterns among the foreign-born have been defined as dietary acculturation [9–11]. This process is characterized by adopting the dietary practices of the dominant food culture, and has been associated with increases in obesity rates among the foreign-born [11–13]. For example, in the US diets may include less fiber, fruits and vegetables, and more meals consumed outside the home compared to the countries of origin [7, 9, 14]. Increasing obesity rates among the foreign-born living in the US have been linked to fast food and eating out [10].

Meals outside the home include breakfast, lunch and dinner purchased or consumed in restaurants or retail outlets, for take-out or delivery. Eating out has been associated with increased calorie intake and weight gain [15, 16]. In fact, compared to eating at home, eating one meal out may increase daily calorie intake by an average of 134 cal. In turn, eating out for just one meal each week could result in an annual weight gain of two pounds leading to significant weight gain over time [15, 16].

However, recent evidence suggests that these dietary changes and associated increase in BMI begin before the foreign-born arrive in the US as a result of the global nutrition transition [12, 14]. Furthermore, study results related to eating out among the foreign-born living in the US differ by geographic location and country of origin. A study among foreign-born Latino parents in California found no association between BMI and meals eaten out regardless of restaurant type. Curiously, in the same study, meals eaten at the homes of friends and relatives were associated with greater calorie intake than restaurant meals or meals eaten at home [17]. Another study examining acculturation among Puerto Rican migrants in Boston found that higher acculturation level was associated with more frequent meals out but also higher dietary quality [18]. Although these studies underscore that there are variations in dietary acculturation across ethnic groups, meals prepared at restaurants did not contribute to obesity risk regardless of population studied.

Changes in eating patterns among the foreign-born occur alongside changes to daily routines [9]. For example, two qualitative studies of African immigrants to New York City (NYC) found that moving to the US meant having less time to cook, eating fast food more often, being less physically activity, and experiencing rapid weight gain [19, 20]. Even so, there is limited population evidence about whether eating out contributes to the risk of obesity among the foreign-born. The two waves (2004 and 2013/14) of NYC HANES data used in this study provide a unique opportunity to detect secular trends in both dietary practices and body size among foreign-born and US-born New Yorkers over the ten-year period.

### Objectives

The purpose of this study was to use the 2004 and 2013/14 waves of the NYC Health and Nutrition Examination Survey (NYC HANES), to compare among foreign-born and US-born New Yorkers by survey year: 1) the odds of obesity among NYC residents; 2) the association between eating out and obesity and 3) whether age at arrival to or duration of residence in the US influenced these relationships among the foreign-born.

Based on previous research we hypothesized that compared to the 2004 cohort, obesity odds would be higher in the 2014 cohort for both the foreign-born and US-born; and that age at arrival and longer duration of residence in the US for the foreign-born would be associated with greater odds of obesity. Furthermore, we hypothesized that eating out would be associated with odds of obesity among the foreign-born in NYC. We examined the effect of eating out on odds of obesity over time by analyzing the two waves of NYC HANES data separately and combined for both the total population, the foreign-born and the US-born.

## Materials and methods

### Study design

NYC HANES is a population based, cross–sectional survey modeled after the National Health and Nutrition Examination Survey (NHANES). Data from the NYC HANES waves 2004 and 2013/14 were used for this study. The sampling methodology randomly selected households within NYC census tracts and then randomly selected eligible adult persons over the age of 20 within the households. The overall response rate for 2004 was 55% (*N* = 1999) and for 2013/14 the response rate was 36% (*N* = 1527). The data collection consisted of 1) an in-person interview, 2) a computer assisted self-interview, and 3) a brief medical exam with biospecimen samples. A more detailed description of the methods is available elsewhere [21, 22]. The NYC HANES 2004 dataset is comprised of 6 separate datasets. Only data from the SPFILE (demographic data), CAPI (nutrition and diet data) and EXAM (medical examination) datasets were used for this analysis. The NYC HANES 2013/14 data are contained in a single dataset. Both 2004 and 2013/14 datasets are de-identified and publicly available, and both had survey weights applied to account for probability of selection and non-response. The two waves of data represent two independent cross sections from different time periods. The datasets have a total sample of 3526 non-institutionalized adult NYC residents aged 20 years and older.

All analyses were weighted to account for the complex sampling survey design, non-response, and post-stratification according to the data analysis guidelines from the New York City Department of Health [21, 22]. Survey participants who were missing data for height and weight (components of BMI) were excluded from the analysis. Thereafter, the weighting for survey participants who had complete data was adjusted by categories of age, sex, and race/ethnicity to ensure that the dataset represented the NYC population. Therefore, all estimates are weighted and representative of the New York City population. The final weighted samples of the 2004 and 2013/14 survey waves represented populations of 5,827,719 and 6,271,280 New Yorkers, respectively.

This secondary data analysis was exempted by the City University of New York institutional ethics review committee.

### Dependent variable

The dichotomized obesity variable was calculated from measured weight and height variables and defined as BMI ≥30 kg/m^2^ or BMI < 30 kg/m^2^. BMI was calculated from measured weight and height and categorized into four categories: underweight BMI < 18.5 kg/m^2^, healthy weight BMI 18.5–24.9 kg/m^2^, overweight BMI 25–29.9 kg/m^2^, and obesity BMI ≥30 kg/m^2^ [23, 24]. We conducted sensitivity analyses and found no difference when comparing underweight + healthy weight to overweight. Therefore, we combined underweight, healthy weight, and overweight. Individual BMI scores and the four BMI classifications were only used for descriptive statistics, whereas the dichotomized obesity variable was the dependent variable in logistic regression models.

### Covariates

Nativity was defined dichotomously as either foreign-born (including US territories) or US-born (including the 50 states and Washington, DC, but excluding US territories). Average weekly consumption of meals away from home was defined as eating out (including breakfast, lunch, or dinner purchased or consumed in restaurants or retail outlets, for take-out or delivery) and standardized across survey years as 0 (never), > 0–1, 2, 3, 4, 5, 6, 7, 8+ times per week. Meals out was constructed from how often participants reported eating out and dichotomized as Yes/No based on the closest whole number to the median in both years (Times per week eating meals out: 2004: Median 0.99; and 2013/14: Median 1.04). Health behavior variables included fruit and vegetable intake, smoking and physical activity. Fruit and vegetable intake, an indicator of dietary quality, was constructed from how often participants reported eating fruits and vegetables and dichotomized as ≥2 times per day vs. < 2 times per day, based on the closest whole number to the median in both years (Times per day eating fruits and vegetables: 2004: Median 2.03; and 2013/14: Median 2.10). For comparison, the United States average daily intake of fruits and vegetables (including potatoes) is 2.5 servings [25]. Smoking was defined as having smoked at least 100 cigarettes and currently smoking every/some days [26]. Physical activity was defined as engaging in moderate and/or vigorous physical activity as part of work, transportation or recreation on average ≥ 10 min/day vs. < 10 min/day [27]. Indicators of acculturation included age at arrival defined as arriving in the US as an adult at ≥18 vs. < 18 years of age, to distinguish between child and adult arrivals. Duration of residence was calculated from age at time of survey and year of arrival in the US and was dichotomized as residing within the US ≥10 years or < 10 years consistent with increased risk of significant weight gain after 10–15 years of residence in the new country [1, 28].

### Independent variables

Sex was collected as female or male. Race and Hispanic/Latino ethnicity were self-reported, and subsequently categorized as non-Hispanic White, non-Hispanic Black, non-Hispanic Asian, Hispanic, and non-Hispanic other. Age categories (20–29, 30–39, 40–49, 50–59, and 60 years and older) were used for descriptive statistics, while respondents reported age (range 20–94) was used for logistic regression models. Income was defined as <$20,000 vs. ≥$20,000. Marital status was defined dichotomously as married or living with a partner vs. never married, separated, or divorced. Education was defined as <high school vs. ≥high school or high school equivalent.

### Statistical methods

We used chi square and t-tests to assess differences between foreign-born and US-born participants in each year and between years. We examined the literature for evidence regarding each specific variable and conducted sensitivity analyses to inform variable composition (including meals out, age at arrival, time in the US, education, income, fruit, and vegetable intake). Tests for collinearity and interaction among the independent variables were conducted before proceeding to weighted multivariate logistic regression.

Weighted logistic regression models estimated adjusted odds ratio (aOR) of obesity by covariates and adjusting for independent variables among the total, foreign-born and US-born populations. Covariates included nativity, eating out, fruit and vegetable consumption, smoking, and physical activity. Independent variables in all models included age, sex, race/ethnicity, education, income, and marital status. Models estimating odds among the foreign-born also included age at arrival and duration of residence in the US.

Model fit to test the global null hypothesis for each model was estimated by second order Rao-Scott Likelihood Ratio Chi-Square Test adjusted for the complex survey design [29, 30]. All models had a Rao-Scott Likelihood Ratio Chi-Square Test *P* < .0001 indicating that the models as a whole fit significantly better than the null model [29, 30].

Six logistic regression models examined the relationship between nativity and obesity: Three models examined differences in obesity odds among all New Yorkers, the foreign-born and the US-born in 2004: Model 1 estimated the relationship between nativity and obesity for the 2004 total sample (Table 2). Model 2 examined the relationship for the 2004 foreign-born population and included age at arrival and duration of residence in the US (Table 2). Model 3 examined the odds of obesity for the 2004 US-born population (Table 2).

Three models examined differences in obesity odds among all New Yorkers, the foreign-born and the US-born in 2013/14. Model 4 estimated the odds of obesity for the 2013/14 total sample (Table 2). Model 5 examined the odds of obesity for the 2013/14 foreign-born sample and included age at arrival and duration of US residence (Table 2). Model 6 examined the odds of obesity for the 2013/14 US-born sample (Table 2).

Three supplementary models examined difference in obesity odds among all New Yorkers, the foreign-born and the US-born between the two survey waves (Additional file 1). Model 1S compared the odds of obesity between 2004 and 2013/14 for the total sample. Model 2S estimated the odds of obesity between 2004 and 2013/14 for the foreign-born only and included age at arrival and duration of US residence. Model 3S estimated the odds of obesity between 2004 and 2013/14 for the US-born only.

All regression models were adjusted for age, sex, race/ethnicity, education, income, marital status. Models estimating odds among the foreign-born also included age at arrival and duration of residence in the US.

All regression analyses accounted for the complex survey design and stratified sampling representative of NYC. Statistical Analysis Software (SAS) version 9.2 was used for analyses.

## Results

Table 1 displays descriptive statistics by survey years.

**Table 1:** Characteristics of the Foreign-Born and US-Born Populations; NYC HANES Years 2004 and 2013/2014

	**A** **NYC HANES 2004**				**B** **NYC HANES 2013/14**				**C** **2004 vs 2013/14**	**D** **2004 vs. 2013/14**
	**Total** **Population Weighted**	**Foreign-** **Born** **Weighted**	**US-** **Born** **Weighted**	***P-*value**^**a**^	**Total** **Population** **Weighted**	**Foreign-Born Weighted**	**US-** **Born Weighted**	***P*-value**^**a**^	**Foreign-** **Born** ***P*-value**^**a**^	**US-** **Born** ***P*-value**^**a**^
	*N* = 1952^b^	*N* = 1073	*N* = 879		*N* = 1482	*N* = 713	*N* = 769			
**Total Population Weighted**^**c**^	100%	52.0%	48.0%		100%	50.0%	50.0%			
**BMI kg/m**^**2**^										
***Obesity, age adjusted*** ^**d**^	26.8%	22.6%	29.3%		31.7%	29.6%	31.9%			
***Mean BMI ± SE***		26.9 *± 0.19*	27.9 *± 0.3*	<.0001		27.7 *± 0.3*	28.1 *± 0.3*	0.3567	<.0001	<.0001
Obesity ≥30 kg/m^2^	26.0%	22.0%	30.2%	<.0001	30.2%	29.8%	30.8%	0.0074	0.0007	0.9680
Overweight, 25–29.9 kg/m^2^	35.3%	39.9%	30.4%		34.4%	38.4%	30.4%			
Healthy Weight 18.5–24.9 kg/m^2^	37.0%	36.7%	37.4%		33.3%	29.4%	37.3%			
Underweight < 18.5 kg/m^2^	1.7%	1.4%	2.0%		2.0%	2.4%	1.7%			
**Eating Out**										
***Mean Times per Week ± SE***	2.50 ± 0.1	2.02 ± 0.1	3.03 ± 0.1	<.0001	2.32 ± 0.1	1.81 ± 0.1	2.85 ± 0.1	<.0001	<.0001	<.0001
No	16.0%	22.5%	8.8%	<.0001	21.5%	33.0%	9.8%	<.0001	0.0006	0.3265
Yes	84.0%	77.5%	91.2%		78.5%	67.0%	90.2%			
**Fruit and Vegetable Intake**										
≥ 2 times per day	55.1%	57.4%	52.7%	0.0452	61.8%	62.9%	60.8%	0.5028	0.0512	0.0054
< 2 times per day	44.9%	42.6%	47.3%		38.2%	37.1%	39.2%			
**Smoking**										
Non-Smoker	76.6%	81.4%	71.3%	<.0001	81.2%	83.9%	78.4%	0.0306	0.2890	0.0080
Smoker	23.4%	18.6%	28.7%		18.8%	16.1%	21.6%			
**Physical Activity ≥ 10 min/day**										
No	44.1%	51.1%	36.5%	<.0001	45.9%	55.8%	35.9%	<.0001	0.0965	0.380
Yes	55.9%	48.9%	63.5%		54.1%	44.2%	64.1%			
**Age at Arrival**										
***Mean ± SE***		28.0 *± 0.5*				25.6 *± 0.82*			0.0017	N/A
< 18 years of age	N/A	22.4%	N/A	N/A	N/A	26.5%	N/A		0.0783	
≥ 18 years of age	N/A	77.6%	N/A	N/A	N/A	73.5%	N/A			
**Time in the US**										
***Mean ± SE***		17.7 *± 0.8*				22.2 *± 0.8*			<.0001	
< 10 years	N/A	33.5%	N/A	N/A	N/A	23.8%	N/A		0.0024	N/A
≥ 10 years	N/A	66.5%	N/A	N/A	N/A	76.2%	N/A			
	**A** **NYC HANES 2004**				**B** **NYC HANES 2013/14**				**C** **2004 vs 2013/14**	**D** **2004 vs. 2013/14**
	**Total** **Population Weighted**	**Foreign-Born** **Weighted**	**US-** **Born** **Weighted**	***P-*value**^**a**^	**Total Population Weighted**	** Foreign-BornWeighted**	**US-Born** **Weighted**	**P-value**^**a**^	***Foreign-Born P-*value**^**a**^	***US-Born P-*value**^**a**^
**Age**										
***Mean ± SE***		45.9 *± 1.0*	45.2 *± 1.0*	<.0001		48.0 *± 0.8*	43.3 *± 0.9*	<.0001	<.0001	<.0001
20–29	19.8%	15.3%	24.7%	<.0001	22.5%	16.3%	28.6%	<.0001	0.0262	0.5895
30–39	22.7%	25.3%	19.8%		19.6%	19.2%	20.3%			
40–49	20.3%	22.5%	17.8%		17.8%	19.2%	16.3%			
50–59	15.6%	16.8%	14.4%		17.0%	19.9%	14.2%			
60+	21.6%	20.1%	23.2%		23.1%	24.5%	20.6%			
**Sex**										
Male	46.1%	46.2%	46.0%		46.7%	44.5%	48.9%	0.1268	0.4631	0.2416
Female	53.9%	53.8%	54.0%	0.9232	53.3%	55.5%	51.1%			
**Ethnicity/Ethnicity**										
Non-Hispanic White	38.2%	21.3%	56.7%	<.0001	35.0%	21.6%	48.7%	<.0001	0.8930	0.0015
Non-Hispanic Black	23.1%	18.1%	28.6%		21.3%	16.8%	25.8%			
Non-Hispanic Asian	10.9%	19.8%	1.2%		14.0%	23.4%	4.5%			
Hispanic	26.2%	39.0%	12.2%		27.1%	36.2%	17.7%			
Non-Hispanic Other	1.5%	1.7%	1.3%		2.6%	1.9%	3.3%			
**Education**										
< High School	27.0%	37.1%	16.2%	<.0001	18.9%	24.6%	13.0%	<.0001	<.0001	0.1907
≥ High School	73.0%	62.9%	83.8%		81.1%	75.4%	87.0%			
**Income**										
≥ $20,000	68.7%	59.5%	78.4%		71.6%	65.9%	77.1%	<.0001	0.0786	0.1634
< $20,000	31.3%	40.5%	21.6%	<.0001	28.4%	34.1%	22.9%			
**Marital Status**										
Married or living w. partner	53.6%	62.0%	44.7%	<.0001	50.9%	60.3%	41.7%	<.0001	0.5914	0.3699
Single, separated, divorced, widowed	46.4%	38.0%	55.3%		49.1%	39.7%	58.2%			

Abbreviations: *SE* standard error, *BMI* Body Mass Index. ^a^ T-tests and χ2 Statistics were used to compare characteristics between foreign-born and US-born New Yorkers

^b^ Sample sizes are unweighted. All other estimates are weighted. ^c^ All weighted proportions reflect New York City population estimates from the American Community Survey 2004 and 2013. Weights include age, gender and race/ethnicity. ^d^ Age adjusted rates were standardized to the US Census 2000 standard population as per NYC HANES data analysis guidelines

### NYC HANES 2004 participants

Among the 2004 total sample, 52% were foreign-born (Table 1, Section A). The top five countries and territories of origin for the foreign-born in the 2004 sample were: Dominican Republic (*n* = 155), China (*n* = 87), Mexico (*n* = 82), Puerto Rico (*n* = 74) and Jamaica (*n* = 43). (Additional file 2).

The foreign-born differed significantly from the US born in nearly all aspects, except for sex. Compared to the US-born, the foreign-born were less likely to have obesity, 22% vs. 30.2%, but more likely to experience overweight, 40% vs. 30.4%, respectively (*P* < 0.0001). Foreign-born were significantly less likely to eat meals away from home compared to the US-born, 75% vs. 91.2% (*P* < 0.0001). The foreign-born were less likely to be smokers (18.6% vs. 28.7%, *P* < 0.0001) and be physically active, as 51.1% reported less than 10 min of physical activity per day compared to a third 36.5% (*P* < 0.0001) of the US-born. A majority (78%) of the foreign-born had arrived in the US at 18 years of age or older and 67% had lived in the US for ≥10 years or more. Foreign-born respondents were more likely to be Hispanic (39% vs. 12.2%), between the ages of 30 and 59 (*P* < 0.0001), to make less than $20 K (40.5% vs. 21.6%, *P* < 0.0001), be married or live with a partner (62% vs. 44.7%, *P* < 0.0001) and have less than a high school education, (37.1% vs. 16.2%), (*P* < 0.0001).

### NYC HANES 2013/14 participants

Of the 2013/14 total sample, 50% were foreign born (Table 1, Section B). The top five countries and territories of origin for the foreign-born in the 2013/14 sample were Dominican Republic (*n* = 81), Puerto Rico (*n* = 63), China (*n* = 58), Jamaica (*n* = 55), and Russia (*n* = 25). (Additional file 2) The foreign-born and US-born differed across all aspects except for sex, and fruit and vegetable intake. The foreign-born were more likely to experience overweight than the US-born (38.4% vs 30.4%), but rates of obesity were similar (29.8% vs. 30.7%), (*P* = 0.0074) and mean BMI was lower (27.7 kg/m^2^ vs. 28.1 kg/m^2^, *P* = < 0.0001). Compared to the US-born, the foreign-born were more likely to never eat meals out (9.8% vs 33%, *P* < 0.0001). The foreign-born were less likely to smoke (*P* = 0.0306), and less physically active, as 44.2% reported engaging in at least 10 min of physical activity per day, compared to 64.1% among the US-born (*P* < 0.0001). Among the foreign-born, a majority had arrived in the US at ≥18 years of age, and 76% had lived in the US for ≥10 years.

Compared to the US-born, the foreign-born were more likely to be Hispanic or Asian (*P* < 0.0001), between the ages of 30 to 60+ years (*P =* 0.0262), more likely to earn <$20 K (*P* < 0.0001), more likely to be married or living with a partner (60.3% vs. 41.7%, *P* < 0.0001), and less likely to have a high school education or above (24.6% vs 13.0%, *P* < 0.0001).

### Participant comparison NYC HANES 2004 vs. 2013/14

The 2013/14 foreign-born sample differed from the 2004 group by having higher mean BMI (*P* < 0.0001), higher obesity rates (*P* = 0.0007), eating out less (*P* = 0.0006), experiencing older age at arrival (*P = 0.0012*) and longer duration of residence (*P* < 0.0001). The 2013/14 US-born differed from the 2004 sample by higher fruit and vegetable intake (*P* = 0.0054), different race/ethnicity (*P* = 0.0015), and lower smoking rates (*P* = 0.008).

### Odds of obesity, NYC HANES 2004

Table 2, Section A displays the logistic regression results from obesity models 1–3. In 2004, foreign-born New Yorkers had lower odds of obesity than the US-born; eating out was not associated with obesity odds in either population. Among the foreign-born obesity odds were higher among those with living in the US ≥10 years.

**Table 2:** Obesity Odds Among Foreign-Born and US-Born New Yorkers, Logistic Regression, Weighted Analysis by Survey Year; New York City Health and Nutrition Examination Survey Years 2004 and 2013/2014

	A NYC HANES 2004^**a**^			B NYC HANES 2013/14^**a**^		
Risk Factors	Obesity aOR (95% CI) Model 1 Total Population	Obesity aOR (95% CI) Model 2 Foreign-Born Only	Obesity aOR (95% CI) Model 3 US-Born Only	Obesity aOR (95% CI) Model 4 Total Population	Obesity aOR (95% CI) Model 5 Foreign-Born Only	Obesity aOR (95% CI) Model 6 US-Born Only
**Nativity**						
US-Born	**Referent**	N/A	N/A	Referent	N/A	N/A
Foreign-Born	0.51 (0.37–0.70)***	N/A	N/A	0.86 (0.61–1.22)	N/A	N/A
**Eating Out**						
No	Referent	Referent	Referent	**Referent**	**Referent**	Referent
Yes	0.93 (0.66–1.34)	0.69 (0.43–1.12)	1.57 (0.88–2.81)	0.64 (0.45–0.90)*	0.49 (0.31–0.77)**	1.18 (0.62–2.25)
**Fruit and Vegetable**						
< 2 times per day	Referent	Referent	Referent	**Referent**	Referent	**Referent**
≥ 2 times per day	0.88 (0.70–1.11)	0.90 (0.62–1.30)	0.85 (0.63–1.16)	0.64 (0.48–0.86)**	0.67 (0.44–1.01)	0.60 (0.40–0.91)*
**Smoking**						
No	Referent	Referent	Referent	Referent	**Referent**	Referent
Yes	0.83 (0.63–1.09)	0.74 (0.45–1.22)	0.76 (0.52–1.13)	0.69 (0.45–1.06)	0.46 (0.23–0.94)*	0.81 (0.48–1.39)
**Physical Activity**						
< 10 Minutes/Day	Referent	Referent	Referent	Referent	Referent	Referent
≥ 10 Minutes/Day	0.88 (0.70–1.12)	1.07 (0.74–1.54)	0.76 (0.52–1.11)	0.97 (0.71–1.34)	1.07 (0.68–1.67)	0.82 (0.55–1.23)
**Age at Arrival**						
< 18 years	N/A	Referent	N/A	N/A	Referent	N/A
≥ 18	N/A	0.76 (0.49–1.20)	N/A	N/A	1.00 (0.56–1.79)	N/A
**Time in the US**						
< 10 years	N/A	Referent	N/A	N/A	Referent	N/A
≥ 10 years	N/A	1.73 (1.08–2.79)*	N/A	N/A	1.53 (0.77–3.04)	N/A
**Age**						
Age, continuous	1.01 (1.01–1.02)***	1.02 (1.00–1.03)	1.01 (1.00–1.02)	1.01 (1.01–1.02)*	1.02 (1.00–1.03)	1.01 (1.00–1.03)
**Sex**						
Male	**Referent**	**Referent**	**Referent**	Referent	Referent	Referent
Female	1.53 (1.22–1.93)***	1.48 (1.01–2.18)*	1.56 (1.13–2.16)**	1.04 (0.76–1.43)	1.23 (0.79–1.93)	0.86 (0.54–1.37)
**Race/Ethnicity**						
Non-Hispanic White	Referent	Referent	Referent	Referent	Referent	Referent
Non-Hispanic Black	1.71 (1.19–2.44)*	0.87 (0.42–1.77)	2.05 (1.34–3.13)	1.64 (1.06–2.54)***	1.29 (0.64–2.62)	1.70 (0.98–2.97)
Hispanic	1.88 (1.30–2.72)**	1.29 (0.76–2.19)	2.23 (1.39–3.57)	1.21 (0.80–1.84)	1.14 (0.65–2.01)	1.37 (0.76–2.46)
Non-Hispanic Asian	0.49 (0.27–0.91)**	0.29 (0.14–0.59)***	2.60 (0.82–8.23)	0.42 (0.21–0.86)**	0.48 (0.20–1.13)	Unreliable *N* = 1
Non-Hispanic Other	1.27 (0.44–3.67)	1.51 (0.36–6.25)	0.56 (0.12–2.66)	0.83 (0.37–1.88)	0.77 (0.18–3.38)	0.84 (0.36–1.95)
**Education**						
≥ High School	Referent	Referent	Referent	Referent	Referent	Referent
< High School	1.54 (1.12–2.11)**	1.50 (1.05–2.17)*	1.60 (1.03–2.49)*	1.56 (1.05–2.31)*	1.05 (0.62–1.79)	2.19 (1.24–3.87)**
**Income**						
$20,000+	Referent	Referent	Referent	Referent	Referent	Referent
< $20,000	1.00 (0.74–1.35)	0.93 (0.66–1.32)	1.21 (0.75–1.98)	0.89 (0.65–1.24)	0.96 (0.57–1.64)	0.87 (0.57–1.32)
**Marital Status**						
Never married, divorced, widowed	Referent	Referent	Referent	Referent	Referent	Referent
Married/Living w. Partner	1.05 (0.84–1.31)	1.22 (0.87–1.70)	1.03 (0.73–1.44)	1.03 (0.75–1.41)	1.20 (0.76–1.89)	0.93 (0.59–1.48)

Significance level: * < 0.05, ** < 0.01, *** < 0.001

Abbreviations: *aOR* Adjusted Odds Ratio

^a^All weighted proportions reflect New York City population estimates. The New York population is weighted by age, gender and race/ethnicity

Model 1 (Table 2, Section A) examined the odds of obesity in the overall NYC HANES 2004 sample. Compared to the US-born, the foreign-born had significantly lower odds of obesity [(OR = 0.51 (95%CI 0.37–0.70) *P* = <.0001]. Age was associated with significantly higher odds of obesity [aOR = 1.01 (95% CI 1.01–1.02) *P* = 0.0006], as was being female [aOR = 1.53 (95% CI 1.22–1.93) *P* = 0.0003], and having less than a high school education [aOR = 1.54 (95% CI 1.12–2.11) *P* = 0.0074]. Compared to non-Hispanic whites, non-Hispanic blacks [aOR = 1.71 (95% CI 1.19–2.44) *P* = 0.0172] and Hispanics [aOR = 1.88 (95% CI 1.30–2.72) *P* = 0.0016] had higher odds of obesity, whereas the non-Hispanic Asian sample had significantly lower odds [aOR = 0.49 (95% CI 0.27–0.91) *P* = 0.0011]. In the 2004 total study sample of foreign-born and US-born; eating out, income, marital status, smoking status, physical activity, and fruit and vegetable intake were not associated with odds of obesity.

Model 2 (Table 2, Section A) examined the odds of obesity among the foreign-born in the NYC HANES 2004 sample only. Duration of residence in the US ≥10 years was associated with 73% higher odds of obesity [aOR = 1.73 (95% CI 1.08–2.79) *P* = 0.0233]. Among the foreign-born, women had 48% higher odds of obesity than men [aOR = 1.48 (95%CI 1.01–2.18) *P* = 0.0466]. Non-Hispanic Asians had lower odds of obesity [aOR = 0.29 (95% CI 0.14–0.59) *P* = 0.0002]. Those with less than a high school education had higher odds of obesity [aOR = 1.50 (95%CI 1.05–2.17) *P* = 0.0283]. Among the foreign-born in 2004 eating out, age at arrival, income, marital status, smoking status, physical activity, and fruit and vegetable intake did not contribute significantly to the odds of obesity.

Model 3 (Table 2, Section A) examined the odds of obesity among the US-born in the NYC HANES 2004 sample only. Among the US-born sample, women had 56% higher odds of obesity [aOR = 1.56 (95%CI 1.13–2.16) *P* = 0.0078]. Respondents with less than high school had higher odds of obesity [aOR = 1.60 (95% CI 1.03–2.49) *P* = 0.0362]. Among the US-born in 2004, eating out, age, race/ethnicity, income, marital status, smoking status, physical activity, and fruit and vegetable intake did not contribute significantly to the odds of obesity.

### Odds of obesity, NYC HANES 2013/14

Table 2, Section B displays the logistic regression results from obesity models 4–6. Overall, in 2013/14 obesity odds were no different between foreign-born and US-born New Yorkers. When examining the two populations separately, eating out was associated with lower odds of obesity among the foreign-born. Eating out was not associated with obesity among the US-born. Time living in the US was not associated with obesity odds among the foreign-born.

Model 4 (Table 2, Section B) examined the odds of obesity in the overall NYC HANES 2013/14 sample. There was no difference in obesity odds between the foreign-born and the US-born. However, eating out was associated with lower odds of obesity [aOR = 0.64 (95% CI 0.45–0.90) *P* = 0.0112]. Each increase in year of age was associated with higher odds of obesity [aOR = 1.01 (95% CI 1.01–1.02) *P* = 0.0016]. When examining obesity odds by race and ethnicity, compared to non-Hispanic Whites, non-Hispanic Blacks had higher odds of obesity [aOR = 1.64 (95% CI 1.06–2.54) *P* = 0.0009] while non-Hispanic Asians had lower odds of obesity [aOR = 0.42 (95% CI 0.21–0.856) *P* = 0.0060]. Those with less than a high school education had higher odds of obesity [aOR = 1.56 (95% CI 1.05–2.31) *P* = 0.0282]. Consuming fruit and vegetables ≥2 times per day was associated with lower odds of obesity [aOR = 0.64 (95% CI 0.48–0.86) *P* = 0.0029]. Among the total sample, sex, income, marital status, smoking and physical activity did not contribute significantly to the odds of obesity. The 2004 NYC HANES did not include information about sugar sweetened beverage intake, but the 2013/2014 survey did. We tested sugar sweetened beverage intake associations and found that this was not associated with either BMI or nativity (data not shown). As such, sugar sweetened beverage intake was not included in the regression models.

Model 5 (Table 2, Section B) examined the odds of obesity among the foreign-born in the NYC HANES 2014 sample only. Among the foreign-born who ate out odds of obesity were less than half [aOR = 0.49 (95%CI 0.31–0.77) *P* = 0.0022]. Smokers had lower obesity odds [aOR = 0.46 (95% CI 0.23–0.94) *P* = 0.0332]*.* Age at arrival, time in the US, age, sex, race/ethnicity, education, income, marital status, physical activity, and fruit and vegetable intake did not contribute significantly to the odds of obesity among the foreign-born in 2013/14.

Model 6 (Table 2, Section B) examined the odds of obesity among the US-born in the NYC HANES 2013/14 sample only. Those with less than a high school education had higher odds of obesity [aOR = 2.19 (95% CI 1.24–3.87) *P* = 0.0074]. Consuming fruit and vegetables ≥2 times per day was associated with lower odds of obesity [aOR = 0.60 (95% CI 0.40–0.91) *P* = 0.0153]. Among the US-born in 2013/14, eating out, age, sex, income, marital status, smoking and physical activity did not contribute significantly to the odds of obesity.

### Odds of obesity, NYC HANES 2004 vs. 2013/14

Overall, for the two survey years, obesity odds were higher for the foreign-born in 2013/14 than in 2004 but odds were not different for the US-born. For foreign-born participants across the two survey years, obesity odds were 42% higher in 2013/14 [aOR = 1.42 (95% CI 1.06–1.89) *P* = 0.0198]. When comparing the US-born participants across the two survey years, obesity odds were no different between 2004 and 2013/14 [aOR = 1.09 (95% CI 0.81–1.47) *P* = 0.5653]. (Additional file 1).

## Discussion

This study sought to determine whether eating out was associated with obesity among foreign-born and US-born New Yorkers and to characterize differences between the 2004 and 2013/14 waves of the NYC HANES. We found that compared to the US-born, the foreign-born had significantly lower odds of obesity in 2004, but similar odds in 2013/14. Although odds of obesity increased among the foreign-born from 2004 to 2013/14, eating out was associated with lower obesity odds among the foreign-born in 2013/14. In contrast, obesity rates and odds remained constant in the US-born population between the two survey years and were not related to eating out. More frequent intake of fruits and vegetables was associated with lower odds of obesity among the US-born. Among the foreign-born, age at arrival was not associated with odds of obesity. Although living in the US ≥10 years was associated with greater odds of obesity in 2004, there was no difference by duration of residence in 2013/14.

The higher obesity prevalence in 2013/14 compared to 2004 appears to be attributable to the increase in obesity among foreign-born New Yorkers alone. Dietary and weight status changes which previously were thought to begin when the foreign-born arrived in the US, may now commence in the home countries such that the foreign-born arrive in the US with more global food preferences and higher mean BMI. Therefore, global food trends may play a larger role in obesity risk among the foreign-born living in the US than how long they have been in the country or the frequency of eating out. The worldwide shift in dietary practices, whereby foods become more affordable, higher in fat, sugar, and processed meat, and lower in fiber is matched with a concurrent decrease in physical activity [12, 31]. This shift may lead to higher rates of obesity and diet-related disease regardless of where people live [32–34].

Immigrants in New York City are more likely to experience overweight than they are to experience obesity. Such findings have been shown in other studies conducted among Filipino [35], African, Caribbean [36, 37] and Hispanic [38, 39] immigrants in New York City. Although race/ethnicity specific analysis was not the focus in this paper, a recent study examining the changes in body mass in the NYC HANES and National Health and Nutrition Examination (NHANES) data demonstrated that weight distribution remained constant among women, but that increases in BMI were observed among men, African Americans, Asians and immigrants without health insurance [39].

The unchanged obesity rates among the US-born over the 10-year study period paired with the protective effect of fruit and vegetable intake may be signs of a slowing obesity epidemic unique to US-born New Yorkers. During the same period, US national age-adjusted obesity prevalence rates increased from 34.6 to 37.9% compared to 30.7% in NYC [8]. The slowing of the obesity epidemic may be due in part to effective public policy and grassroots efforts in NYC. Over the past three decades, NYC has been intentional in crafting food policy initiatives to address overall health, obesity and improve quality, quantity, and accessibility of healthy foods for all New Yorkers. Examples include: 1) the passage of menu calorie labeling legislation for chain restaurants implemented in 2006 [40, 41]; 2) the strategic placement of mobile fruit and vegetable vendors in underserved neighborhoods [42]; 3) the Food Retail Expansion to Support Health (FRESH) program which has coordinated rezoning, tax incentivization and strategic opening of 18 supermarkets in food deserts since 2009 [43]; 4) a nutrition education marketing campaign by the NYC Department of Health focused on reducing sugar sweetened beverage intake [44]; and 5) concerted efforts by non-profit organizations and the NYC DOH to implement city, state and federally funded nutrition education programs targeting New Yorkers of all ages in childcare, Head Start, WIC, schools, after-school, senior programs and community-based organizations [45, 46].

We had hypothesized that eating out would be associated with odds of obesity among the foreign-born, but we did not anticipate that it would be associated with lower odds of obesity among this sample. The negative association between meals away from home and obesity is unlikely to be an indication of “protection” against obesity such that eating out might be interpreted as “healthy.” Still, the findings stand in contrast to previous research and demands further examination. The association may represent a convergence of other factors which influence the health behaviors of foreign-born New Yorkers [15, 47]. In fact, Americans of all walks of life are less likely to cook dinner than in years past. Importantly, despite the perception that meals prepared at home are healthier, the most commonly foods reported eaten for dinner are cereal, toaster pastries, yogurt and tap water [48–50]. However, dietary quality and frequency of meals prepared at home may depend on diverse factors such as income, food availability, time constraints and incorporation of processed ingredients [14, 51]. Rising cost of living in NYC has forced many to move further away from employment centers which has increased travel and time away from home further reducing time available to cook and eat at home [52, 53].

Although immigrants living in NYC may prefer home-cooked meals and culturally specific foods [19, 48, 54], they may not always have the opportunity [19, 48, 54]. Long commutes, working multiple jobs, and lack of access to home kitchen facilities may dictate where, when and what kinds of meals are prepared and consumed [47, 48]. A study examining NHANES Data (2007–2008) compared US-born and foreign-born participants with low education and low income. They found that the foreign-born were significantly more likely to report either cooking 6–7 dinners at home per week or never cooking dinner at home [47]. Based on market research, consumers are seeking fast, healthy, fully prepared and fresh dinner products to pick up at grocery stores and eat at home [49]. In response, food retail and supermarket industries are responding with freshly prepared meals ready for purchase. Unfortunately, the NYC HANES questions about meals do not provide information about location or quality.

For both the foreign-born and US-born, NYC offers opportunities to purchase a variety of cultural foods from take-out restaurants and mobile food vendors in walkable neighborhoods. However, the healthfulness of these meals remains largely undocumented [55, 56]. A recent study from Los Angeles documented that the healthy immigrant effect in cultural enclaves with high concentrations of foreign-born residents offer healthier options and better access to fresh fruits and vegetables that extend to the US-born [57]. Acknowledging that New Yorkers may rely on more meals prepared away from home, future interventions might focus on enhancing meal options in neighborhood restaurants, take-out places and supermarkets [58]. NYC food policy efforts have already demonstrated that meals offered by chain restaurants may become healthier in response to requiring more transparency related to calorie labeling, serving sizes, and ingredients such as trans-fats and preparation methods [40, 41, 59, 60].

Contrary to previous findings in foreign-born populations living in the US [4, 61, 62], this study did not find an association between age at arrival and obesity. This may indicate that the foreign-born are becoming more similar to the US-born regardless of age at arrival. The increasing obesity rates in the low- and middle-income countries of origin of many NYC foreign-born inhabitants may reflect a global food system providing more affordable, processed and energy-dense food [63].

Duration of residence was included in this study because it has been used in past studies as a proxy for dietary acculturation. The longer someone resides in the US, the more likely they are to adopt dominant food practices [1, 4, 56, 61, 64–67]. Indeed, in our study in 2004 living in the US ≥10 years was linked to higher odds of obesity compared to those who had resided in the US for less time. Nevertheless, in the 2013/14, duration of residence was no longer a predictor of obesity suggesting a narrowing of the obesity gap between more recent arrivals and those who have been in the country for at least a decade. However, due to the cross-sectional data used in this and other studies examining duration of residence, the direct effect of time spent in the US is less clear [9, 32, 33].

### Strengths and limitations

The NYC HANES data includes both self-reported and clinically assessed data related to obesity. Detailed sociodemographic and health behavior information allows for meaningful exploration of associations. However, NYC HANES is a cross sectional survey which does not allow for causal inferences. Nevertheless, the use of a combined NYC HANES 2004 and 2013/14 dataset is valuable because of its representativeness, large sample of the NYC population, and the detailed data available for each participant. The NYC HANES data offers an opportunity to examine differences over time. However, the two cross-sectional samples do not allow us to distinguish between differences related to the samples and changes to obesity which have occurred over the last 10 years among those arriving in the US.

There are limitations to this study. The NYC HANES 2013/14 had a lower response rate than NYC HANES 2004. Response rates have been declining nationally [68–71]. The NYC HANES survey may be perceived as particularly burdensome to complete since it requires a two-part interview (in-person and computer administered surveys), a physical examination and biospecimen collection [21, 22]. The residents who agree to participate may not reflect their actual representation in the city’s population. In turn, the sample was weighted by the American Community Survey 2004 and 2013 population data to reflect the NYC population. Due to the smaller sample size in the 2013/14 NYC HANES survey all analyses should be interpreted with caution; the results may not be generalizable beyond NYC. Nevertheless, this study remains the only large scale study that contains both behavioral and clinical measurements of the diverse US- and foreign-born New York City population.

Second, we recognize that categorizing populations by nativity without acknowledging racial/ethnic, cultural identities or geographic region of origin obscures differences between sub-groups within both the foreign-born and US-born [72]. It is possible that the overall negative association between obesity and eating out masks heterogeneous effects that differ by race/ethnicity. Unfortunately, race/ethnicity numbers are not sufficiently large to allow for this testing. Future studies should examine specific population groups independently to better assess variations within the foreign-born and US-born groups.

Third, this paper focused on obesity as the outcome, however, overweight rates among the foreign-born also increased over the ten-year period. While overweight was not the focus of this study it may be grounds for future research to examine whether and how rapidly New Yorkers transition from the overweight BMI category to obesity. However, such analysis is beyond NYC HANES cross sectional data.

Fourth, this study does not include information about the nutritional content of meals based on where they were prepared and/or consumed. Therefore, to gain a better understanding of dietary quality associated with meal settings, future dietary assessment surveys should include information about meal settings, frequency, and volume of food as well as preparation methods. Although sugar sweetened beverages have been shown to be higher among immigrants to the US and may contribute to obesity risk both among immigrants and the US-born [38, 73], sugar sweetened beverage intake was not included in the 2004 dataset and we were therefore unable to include it in the comparison.

## Conclusions

This study demonstrates that obesity odds are no longer different between foreign-born and US-born New Yorkers. Higher obesity odds among foreign-born New Yorkers are not explained by age at arrival, or duration of residence. The foreign-born are less likely to eat out than the US-born, but this practice does not correspond to odds of obesity even when controlling for sociodemographic, and health behaviors. In fact, eating out is associated with lower odds of obesity among the foreign-born. Understanding the underlying causes of weight status among the foreign-born and US-born and how these contribute to health outcomes is important to prevent an increase in cardiometabolic diseases. Future research should examine the global and local contextual factors associated with the increase in obesity prevalence as well as expand our understanding of individual-level frequency, locations, quality, and quantity of meals eaten both inside and outside the home among both the foreign-born and US-born living in NYC.

## Supplementary Information

**Additional file 1.** Obesity and Nativity Pooled Analysis NYC HANES 2004 and 2013/14.

**Additional file 2.** Countries of Origin NYC HANES 2004 and 2013/14/.

**Additional file 3.** Crude Regression Models NYC HANES 2004 and 2013/14.

## Data Availability

The NYC HANES data is publicly available from the NYC Department of Health and Mental Hygiene: http://nychanes.org/
