# Supplementary material for: Increasing obesity odds among foreign-born New Yorkers are not explained by eating out, age at arrival, or duration of residence: results from NYC HANES 2004 and 2013/2014
Source: BMC Public Health. 2021 Jul 26;21:1453. doi: 10.1186/s12889-021-11351-1 (PMC8311945; doi:10.1186/s12889-021-11351-1)
Supplement: Supplementary file 1 — Additional file 1. Obesity and Nativity Pooled Analysis NYC HANES 2004 and 2013/14. [file 12889_2021_11351_MOESM1_ESM.docx]

Increasing Obesity Odds Among Foreign-Born New Yorkers are Not Explained by Eating out, Age at Arrival, or Duration of Residence: Results from NYC HANES 2004 and 2013/2014

Margrethe F. Horlyck-Romanovsky^1^ and Sean J. Haley^2^

Affiliations:

^1^ Department of Health and Nutrition Sciences, Brooklyn College, City University of New York, 2900 Bedford Avenue, Brooklyn, NY

^2^ Department of Health Policy and Management, CUNY Graduate School of Public Health and Health Policy, 55 West 125^th^ Street, New York, NY

Corresponding author:

Margrethe F. Horlyck-Romanovsky ORCID: 0000-0002-2146-2744

Department of Health and Nutrition Sciences, Brooklyn College

City University of New York

2900 Bedford Avenue

Brooklyn, NY 11210

[MargretheHR@brooklyn.cuny.edu](mailto:MargretheHR@brooklyn.cuny.edu)

718-951-2753

**Proposed Running head:** Nativity and Obesity, NYC HANES 2004 and 2013/14

**Additional File 1. Comparison of Obesity Odds for Foreign-Born and US-Born, Logistic Regression, Weighted Pooled New York City Health and Nutrition Examination Survey Years 2004 and 2013/2014.**

|  | **Pooled Population Sample NYC HANES 2004 and 2014** | | |
| --- | --- | --- | --- |
| **Risk Factors** | **1S**  **Obesity**  **aOR (95% CI)**  **Model 1S**  **Total Population** | **2S**  **Obesity**  **aOR (95% CI)**  **Model 2S**  **Foreign-Born Only** | **3S**  **Obesity**  **aOR (95% CI) Model 3S**  **US-Born Only** |
| **Survey Year** |  |  |  |
| 2004 | Referent | Referent | Referent |
| 2013/14 | **1.28 (1.05-1.55)*** | **1.42 (1.06-1.89)*** | 1.09 (0.81-1.47) |
| **Nativity** |  |  |  |
| US-Born | Referent | N/A | N/A |
| Foreign-Born | **0.67 (0.53-0.85)**** | N/A | N/A |
| **Eating Out** |  |  |  |
| No | Referent | Referent | Referent |
| Yes | **0.75 (0.59-0.97)*** | **0.59 (0.42-0.81)**** | 1.36 (0.88-2.11) |
| **Fruit and Vegetable** |  |  |  |
| <2 times per day | Referent | Referent | Referent |
| ≥2 times per day | **0.75 (0.62-0.91)**** | 0.77 (0.58-1.01) | **0.74 (0.57-0.96)*** |
| **Smoking** |  |  |  |
| No | Referent | Referent | Referent |
| Yes | 0.79 (0.62-1.01) | **0.61 (0.41-0.92)*** | 0.80 (0.59-1.09) |
| **Physical Activity** |  |  |  |
| <10 Minutes/Day | Referent | Referent | Referent |
| ≥10 Minutes/Day | 0.91 (0.75-1.11) | 1.07 (0.80-1.43) | 0.77 (0.59-1.00) |
| **Age at Arrival** |  |  |  |
| <18 years | N/A | Referent | N/A |
| ≥18 | N/A | 0.90 (0.61-1.32) | N/A |
| **Time in the US** |  |  |  |
| <10 years | N/A | Referent | N/A |
| ≥10 years | N/A | **1.64 (1.11-2.42)*** | N/A |
| **Age** |  |  |  |
| Age, continuous | **1.01 (1.01-1.02)***** | **1.02 (1.04-1.03)**** | **1.01 (1.00-1.02)*** |
| **Sex** |  |  |  |
| Male | Referent | Referent | Referent |
| Female | **1.28 (1.04-1.56)*** | **1.37 (1.03-1.81)*** | 1.16 (0.87-1.57) |
| **Race/Ethnicity** |  |  |  |
| Non-Hispanic White | Referent | Referent | Referent |
| Non-Hispanic Black | **1.65 (1.26-2.16)***** | 1.07 (0.65-1.77) | **1.83 (1.32-2.55)***** |
| Hispanic | **1.50 (1.14-1.96)***** | **1.23 (0.84-1.80)*** | **1.73 (1.19-2.52)***** |
| Non-Hispanic Asian | **0.46 (0.28-0.75)***** | **0.40 (0.22-0.73)**** | **0.30 (0.11-0.82)**** |
| Non-Hispanic Other | 0.98 (0.53-1.79) | 1.01 (0.34-2.98) | 0.79 (0.39-1.59) |
| **Education** |  |  |  |
| ≥High School | Referent | Referent | Referent |
| <High School | **1.53 (1.20-1.97)***** | 1.31 (0.95-1.79) | **1.86 (1.31-2.65)***** |
| **Income** |  |  |  |
| ≥$20,000 | Referent | Referent | Referent |
| <$20,000 | 0.94 (0.75-1.17) | 0.94 (0.69-1.28) | 1.04 (0.76-1.44) |
| **Marital Status** |  |  |  |
| Never married, divorced, widowed | Referent | Referent | Referent |
| Married/Living w. Partner | 1.05 (0.86-1.27) | 1.24 (0.93-1.64) | 0.97 (0.74-1.28) |

Significance level: * <0.05, **<0.01, ***<0.001
