# Supplementary material for: Increasing obesity odds among foreign-born New Yorkers are not explained by eating out, age at arrival, or duration of residence: results from NYC HANES 2004 and 2013/2014
Source: BMC Public Health. 2021 Jul 26;21:1453. doi: 10.1186/s12889-021-11351-1 (PMC8311945; doi:10.1186/s12889-021-11351-1)
Supplement: Supplementary file 2 — Additional file 2. Countries of Origin NYC HANES 2004 and 2013/14/. [file 12889_2021_11351_MOESM2_ESM.pdf]

Additional File 2. Countries of Origin for Foreign-Born New Yorkers, NYC HANES 2004 and 2023/20214 (excerpts of codebooks)

VARIABLE NAME: DMQ105 **SAS FORMAT NAME: DMQ105F.**
SAS Label: Country of birth coded
Question: In what country {were you/was SP} born?
Instructions: DO NOT READ LIST OF COUNTRIES OUT LOUD TO RESPONDENT

Value	Value Label	Frequency	Cumulative Frequency	Percent	Weighted Percent	Skip to
10	United States	898	898	44.92	47.92	DMQ130e
11	Puerto Rico	74	972	3.70	3.10	DMQ161M
12	Dominican Republic	155	1127	7.75	6.02	DMQ161M
13	Jamaica	43	1170	2.15	2.38	DMQ161M
14	Mexico	82	1252	4.10	3.03	DMQ161M
15	China	87	1339	4.35	3.32	DMQ161M
16	Russia	30	1369	1.50	1.97	DMQ161M
66	Other	623	1992	31.17	31.91	
77	Refused	1	1993	0.05	0.05	DMQ161M

Not Asked or Missing = 6

https://www1.nyc.gov/assets/doh/downloads/pdf/hanes/codebook_spfile.pdf

NYC HANES NYC Health CUNY SPH 5

New York City Health and Nutrition Survey (NYC HANES) 2013-14 Codebook - PUBLIC DATASET

Section	Raw Name	Question	Format	2004 Study	Notes	Mean	Value	Frequency (unweighted)	Percent (unweighted)	Percent (weighted)
DMQ	DMQ_5	In what country was SP born?	$COUNTRY	DMQ105	Ask all		10: United States	787	51.54	49.43
							11: Puerto Rico	63	4.13	4.06
							12: Dominican Republic	81	5.30	5.38
							13: Jamaica	55	3.60	3.62
							14: Mexico	24	1.57	2.08
							15: China	58	3.80	4.19
							16: Russia	25	1.64	1.83
							17: Guyana	22	1.44	1.26
							18: Ecuador	21	1.38	1.58
							19: Haiti	13	0.85	0.73
							20: India	15	0.98	1.00
							21: Korea	16	1.05	0.90
							22: Trinidad and Tobago	24	1.57	1.70
							23: Colombia	19	1.24	1.41
							24: United Kingdom	9	0.59	0.55
							25: Philippines	18	1.18	1.22
							26: Italy	6	0.39	0.47
							27: Ireland	4	0.26	0.34
							28: Japan	3	0.20	0.16
							29: Ukraine	20	1.31	1.53
							30: Germany	6	0.39	0.44
							66: Other	230	15.06	15.50
							DN: Don't know	4	0.26	0.32
							R: Refusal	4	0.26	0.32

http://nychanes.org/wp-content/uploads/sites/6/2019/06/28283961_NYC-HANES_codebook_Public_V3_051419.pdf
