# Supplementary material for: Increasing obesity odds among foreign-born New Yorkers are not explained by eating out, age at arrival, or duration of residence: results from NYC HANES 2004 and 2013/2014
Source: BMC Public Health. 2021 Jul 26;21:1453. doi: 10.1186/s12889-021-11351-1 (PMC8311945; doi:10.1186/s12889-021-11351-1)
Supplement: Supplementary file 3 — Additional file 3. Crude Regression Models NYC HANES 2004 and 2013/14. [file 12889_2021_11351_MOESM3_ESM.docx]

**Additional File 3. Obesity Odds Among Foreign-Born and US-Born New Yorkers, Logistic Regression, Weighted Analysis by Survey Year; New York City Health and Nutrition Examination Survey Years 2004 and 2013/2014.**

|  | **A**  **NYC HANES 2004^a^** | | | **B**  **NYC HANES 2013/14^a^** | | |
| --- | --- | --- | --- | --- | --- | --- |
| **Risk Factors** | **Obesity**  **OR (95% CI)**  **Crude Models**  **Total Population** | **Obesity**  **OR (95% CI) Crude Models**  **Foreign-Born Only** | **Obesity**  **OR (95% CI) Crude Models**  **US-Born Only** | **Obesity**  **OR (95% CI) Crude Models**  **Total Population** | **Obesity**  **OR (95% CI) Crude Models**  **Foreign-Born Only** | **Obesity**  **OR (95% CI) Crude Models**  **US-Born Only** |
| **Nativity** |  |  |  |  |  |  |
| US-Born | **Referent** | N/A | N/A | Referent | N/A | N/A |
| Foreign-Born | **0.65 (0.51-0.83)***** | N/A | N/A | 0.95 (0.74-1.23) | N/A | N/A |
|  |  |  |  |  |  |  |
| **Eating Out** |  |  |  |  |  |  |
| No | Referent | **Referent** | Referent | **Referent** | **Referent** | Referent |
| Yes | 0.80 (0.57-1.12) | **0.58 (0.39-0.88)**** | 1.03 (0.58-1.84) | **0.66 (0.50-0.86)***** | **0.51 (0.36-0.72)***** | 1.07 (0.63-1.83) |
|  |  |  |  |  |  |  |
| **Fruit and Vegetable** |  |  |  |  |  |  |
| <2 times per day | Referent | Referent | Referent | **Referent** | **Referent** | **Referent** |
| ≥2 times per day | 0.90 (0.73-1.11) | 0.96 (0.70-1.32) | 0.88 (0.66-1.17) | **0.57 (0.44-0.74)***** | **0.62 (0.43-0.89)**** | **0.55 (0.39-0.77)**** |
|  |  |  |  |  |  |  |
| **Smoking** |  |  |  |  |  |  |
| No | Referent | **Referent** | Referent | Referent | Referent | Referent |
| Yes | 0.86 (0.67-1.11) | **0.60 (0.40-0.91)*** | 0.95 (0.67-1.34) | 0.71 (0.51-1.00) | 0.50 (0.28-0.91) | 0.92 (0.61-1.38) |
|  |  |  |  |  |  |  |
| **Physical Activity** |  |  |  |  |  |  |
| <10 Minutes/Day | Referent | Referent | **Referent** | **Referent** | Referent | **Referent** |
| ≥10 Minutes/Day | 0.85 (0.67-1.07) | 0.97 (0.68-1.37) | **0.65 (0.46-0.91)*** | **0.76 (0.57-0.99)*** | 0.87 (0.60-1.25) | **0.65 (0.45-0.96)*** |
|  |  |  |  |  |  |  |
| **Age at Arrival** |  |  |  |  |  |  |
| <18 years | N/A | Referent | N/A | N/A | Referent | N/A |
| ≥18 | N/A | 0.85 (0.58-1.24) | N/A | N/A | 1.13 (0.74-1.71) | N/A |
|  |  |  |  |  |  |  |
| **Time in the US** |  |  |  |  |  |  |
| <10 years | N/A | **Referent** | N/A | N/A | **Referent** | N/A |
| ≥10 years | N/A | **2.44 (1.69-3.54)***** | N/A | N/A | **2.02 (1.31-3.13)**** | N/A |
| Significance level: * <0.05, **<0.01, ***<0.001  Abbreviations: OR: Odds Ratio  **^a^**All weighted proportions reflect New York City population estimates. The New York population is weighted by age, gender and race/ethnicity, but unadjusted. | | | | | | |
